# Effect of dietary protein level and lamb breed on meat physicochemical traits, fatty acid profile and nutritional indices

**DOI:** 10.5194/aab-68-57-2025

**Published:** 2025-01-30

**Authors:** Hadhami Hajji, Samir Smeti, Ilyes Mekki, Naziha Atti

**Affiliations:** 1 University of Carthage, National Institute of Agronomic Research of Tunisia, Animal and Forage Productions Laboratory, Rue Hédi Karray, 2049 Ariana, Tunisia; 2 University of Gabès, Livestock and Wildlife Laboratory, Arid Regions Institute (IRA), 4119 Medenine, Tunisia

## Abstract

Special attention is increasingly given to food characteristics, particularly fatty acid (FA) profile. The quality of meat, as food of animal origin, depends on animal genotype and feeding. This study evaluated the meat quality and FA profile of three Tunisian sheep breeds, i.e. Barbarine (BB), Queue Fine de l'Ouest (QFO) and Noire de Thibar (NT), under diets with low (11 %) or high (16 %) crude protein (CP) levels, aiming to optimize feeding strategies for Tunisian sheep production. Lambs were slaughtered at 51 kg body weight. The longissimus-thoracis et lumborum muscle was extracted for meat quality analysis.

The intramuscular fat was higher for QFO than other breeds. The meat FA profile was not affected by CP level but was affected by sheep breed. The C16:0 FA was higher for QFO than the two other breeds, which had higher C18:0 FA (17.7 vs. 14.6 %). The n-3 and n-6 polyunsaturated FA (PUFA) contents were higher ( P<0.001 ) for meat of fat-tailed BB than other breeds, resulting in a higher total PUFA and higher PUFA / SFA ratio (0.135 vs. 0.09, where SFA represents saturated fatty acid). The atherogenic index (AI) and cholesterolemic index ( h/H ) of the meat were in the recommended ranges, being in favour of BB and NT breeds with, respectively, lower and higher values than QFO. However, the thrombogenic index (TI) was high and similar among breeds. In conclusion, the FA profile and lipid indices in sheep meat were primarily influenced by breed, with no significant effect from crude protein level, suggesting that a low-protein diet can yield comparable meat quality to a high-protein diet.

## 1 Introduction

Nutritious foods, which provide good health and welfare for humans, are nowadays researched and have a particular consideration by consumers the world over. Special attention is increasingly given to the fatty acid (FA) profile of food and its partition into saturated fatty acids (SFAs) and polyunsaturated fatty acids (PUFAs) in relationship with their effects and impacts on human health (Roopashree et al., 2021). As food, the meat of lamb is widely appreciated by consumers in the Mediterranean area (Smeti et al., 2014; Krajnc et al., 2021). However, meat quality is subject to many factors affecting animals in pre-mortem and post-mortem phases. The main reported differences related to the animal feeding system are in carcass fatness (Majdoub-Mathlouthi et al., 2013; Yagoubi et al., 2018), meat colour (Smeti et al., 2014; Turner et al., 2014) and fatty acid composition (Aksoy et al., 2019; Echegaray et al., 2021a). Furthermore, the animal, as a production unit characterized by its breed, age and slaughter weight, can affect these qualitative traits. The breed evaluation experiments provided deep knowledge about breed divergence and revealed essential information for effective development and use of genetic resources (Echegaray et al., 2021b). For sheep production, there are two main breed types, fat-tailed sheep and tailed sheep with thin tails. To our knowledge, published data comparing meat quality parameters and FA profiles of lambs from fat-tailed and thin-tailed breeds are scare (Yousefi et al., 2012). On the other hand, the effects of energetic level and source of n-3 PUFAs on the meat FA profile (De Smet et al., 2000; Atti et al., 2005) as well as protein source (Łozicki et al., 2013) were published, while the effects of the crude protein (CP) level on these parameters (meat quality and FA profile) were not published. Considering these factors, we undertook this experiment with the aim of evaluating and comparing the meat quality traits, especially the fatty acid (FA) profile, of three Tunisian sheep breeds: one fat-tailed breed (Barbarine (BB)) and two thin-tailed breeds (Queue Fine de l'Ouest (QFO) and Noire de Thibar (NT)). These breeds are of particular relevance within the Tunisian context due to their adaptability to local conditions and their significance in traditional livestock systems. By subjecting lambs from these breeds to two levels of dietary crude protein (CP), i.e. a low-protein diet (11 %) and a high-protein diet (16 %), this study aims to provide insights into how protein intake levels and lamb breed influence meat quality parameters and FA profiles. This work holds particular importance for Tunisia's sheep production sector, as it can inform sustainable feeding strategies. By evaluating the potential for lower-protein diets to maintain meat quality, the study seeks to support local producers in optimizing resources while meeting consumer demands for high-quality, health-beneficial meat. The findings will contribute valuable knowledge to enhance breed-specific feeding recommendations, ultimately benefiting both local sheep producers and consumers.

## 2 Material and methods

### 2.1 Experimental design and meat sampling

A total of 42 lambs were used in this study, belonging to three different breeds: one fat-tailed breed (Barbarine (BB)) and two thin-tailed breeds (Queue Fine de l'Ouest (QFO) and Noire de Thibar (NT)). Each breed consisted of 14 lambs, which were divided into two diet groups (seven lambs per group) based on dietary crude protein (CP) levels: a low-protein (LP) group (110 g kg^−1^ of CP dry matter) and a high-protein (HP) group (160 g kg^−1^ of CP dry matter). The diet consisted of a mix of oat hay and concentrate, with a forage-to-concentrate ratio of 1:2 . The initial body weight (BW) of lambs was 32.1 kg, and the final BW at slaughter after 120 d was 50 kg. One-hour post-mortem, the carcasses were placed in a refrigerated room at 4 °C air temperature for 24 h. Then they were split longitudinally in half, and the left longissimus thoracis et lumborum (LTL) muscle was excised from each carcass. Raw meat samples, taken from the LTL muscle, were trimmed of external fat and divided into five samples, two of them were immediately used for pH, colour parameters and water cooking loss determination. One sample was dried by lyophilization, ground (1 mm screen) and stored for subsequent chemical analysis (ash, CP and fat). The last two samples were frozen at - 20 °C for sensorial quality and individual fatty acid (FA) determination.

### 2.2 Physical and chemical analyses

The pH was measured 1 and 24 h post-mortem with a penetrating electrode connected to a portable pH meter (Hanna Instruments HI 99163, Romania) after calibration with two buffers (7.01 and 4.01). For water cooking loss determination, meat samples of the LTL muscle were weighed (initial weight, $W_{i}$ ), held in plastic bags, immersed in a water bath at 75 °C, and heated for 30 min until the internal temperature reached 75 °C (monitored with a thermocouple). Then the bags were cooled under running tap water for 30 min and blotted dry with paper towels. The cooked meat was weighed again (final weight, $W_{f}$ ), and cooking loss (g kg^−1^) was calculated as 1000 × ( $W_{i}$ - $W_{f}$ ) / $W_{i}$ .

For meat colour parameters, a Minolta CM-2006d spectrophotometer (Konica Minolta Holdings, Inc, Osaka, Japan) was used to measure colour directly on the muscle surface; the measured area diameter was 8 mm. International Commission on Illumination (CIE) $L^{*}$ (lightness), $a^{*}$ (redness) and $b^{*}$ (yellowness) parameters were recorded. $H^{*}$ (hue angle) and $C^{*}$ (chroma) indices were calculated as $H^{*}$ = $\mathrm{tan}-1(b^{*}/a^{*})\times 57.29$ , expressed in degrees, and $C^{*}$ = $(a^{*}2+b^{*}2)1/2$ . $H^{*}$ is the attribute of a colour perception denoted by blue, green, yellow, red, purple, etc; $C^{*}$ is related to the quantity of pigments: high values represent a more vivid colour and denote lack of greyness.

For meat chemical composition, ground meat samples were used. The ash content was determined as the residue after combustion at 600 °C for 8 h. Total nitrogen was determined by the Kjeldahl method using a Büchi Digestion Automat K-438 and Büchi Distillation Unit B-324 (Büchi Laboratory Equipment, Flawil, Switzerland). The CP was calculated as N×6.25 . Intramuscular fat (IMF) was extracted using an automated Soxhlet extractor with hexane as solvent (AOAC, 1999).

### 2.3 Lipid extraction, methylation and fatty acid analysis

Intramuscular fat extraction was carried out according to the Bligh and Dyer (1959) method with the following modifications: 2.5 g of lyophilized minced samples of muscle was mixed with 5 mL of chloroform and 10 mL of methanol and vortexed for 2 min. Another 5 mL of chloroform and 10 mL of 0.88 % KCl were added, vortexed during 15 min and centrifuged at 4000 rpm (revolutions per minute) for 10 min at - 4 °C. The lower phase (FA and chloroform) was extracted and deposited into a glass tube with 10 µ L butylated hydroxytoluene (BHT). A rotary evaporator was used to dry the fat extracts under vacuum, and the extracts were dried in a vacuum oven at 50 °C. The extracted fat was stored in a Polytop (glass vial, with push-in top) and stored at - 80 °C until further analyses.

Meat FA composition was determined by capillary gas chromatography of the fatty acid methyl esters (FAMEs). These FAMEs were prepared by base-catalysed methanolysis of the glycerides with KOH according to the UNE-EN ISO 5509:2000 method. FAMEs were separated and determined using a gas chromatograph with flame ionization detector (GC-FID, Bruker 436-gas chromatograph) equipped with a capillary column of biscyanopropyl polisiloxane (BR2560 WCOT 100 m, 0.25 mm i.d. and 0.20 m film thickness; Bruker Chemical Analysis B.V., the Netherlands). The carrier gas was helium, and the flow rate was 1 mL min^−1^. The temperatures of the inlet and detector were maintained at 250 and 275 °C, respectively. The injection volume was 1.0 mL (split 1:50 ). The temperature programme was as follows: the initial temperature was held at 140 °C after injection, then programmed to increase by 3.0 °C min^−1^ to reach 170 °C (held there for 10 min), then increased by 1.0 °C min^−1^ to reach 180 °C (held there for 5 min), then after that increased at 20.0 °C min^−1^ to attain 210 °C (held there for 24 min) and finally increased by 30 °C min^−1^ to reach 220 °C (held there for 15 min). Analysis time was 78.50 min (including equilibration time). FAME identification was based on retention times as compared with those of the standard FAME mixture.

Individual FA contents were expressed as percentages of total FA. The atherogenic index (AI), thrombogenic index (TI) and the health-promoting index (HPI) as well as the ratio of hypocholesterolemic / hypercholesterolemic FA ( H/h ) were calculated:

$$\begin{array}{rl} & \mathrm{AI}=\left(\text{C12:0}+4\text{C14:0}+C16:0\right)\\ & \;/\left(\sum \mathrm{MUFA}+\sum \mathrm{PUFA}\right);\\ & \;\left(\mathrm{MUFA}:\mathrm{monounsaturated}\;\;\mathrm{FA}\right),\\ & \mathrm{TI}=\left(\text{C14:0}+\text{C16:0}+\text{C18:0}\right)/[\left(0.5\times \Sigma \mathrm{MUFA}\right)\\ & +\left(0.5\times \Sigma \mathrm{PUFAn}6\right)+\left(3\times \Sigma \mathrm{PUFAn}3\right)+\left(\Sigma n3/\Sigma n6\right)],\\ & \mathrm{HPI}=\Sigma \mathrm{UFA}/\left[\text{C12:0}+\left(4\times \text{C14:0}\right)+\text{C16:0}\right];\\ & \;\left(\mathrm{UFA}:\mathrm{unsaturated}\;\;\mathrm{FA}\right)\\ & h/H=\left(cis\text{-C18:1}+\Sigma \mathrm{PUFA}\right)/\left(\text{C14:0}+\text{C16:0}\right). \end{array}$$

### 2.4 Sensory analysis

A sensory evaluation panel with 15 trained members was used to evaluate the meat sensory characteristics. Unsalted meat samples were roasted in aluminium foil in a pre-heated oven at 180 °C for 30 min. Each sample was cut into five pieces of 1×1 cm, and each piece was labelled and then served in random order. Panellists rated meat sample for different attributes on a nine-point hedonic scale corresponding to the intensity of their different feelings for each attribute as follows: 1 = very low intensity, 3 = low intensity, 5 = medium intensity, 7 = intensive and 9 = very intensive. Bread and water were provided for the trained panellists to cleanse the palate between every two samples.

### 2.5 Statistical analysis

Statistical analysis was performed by analysis of variance using the general linear model (GLM) procedure of SAS software (1999). The effects of dietary treatment (CP level), breed and their interaction on different meat quality parameters were analysed according to the following model:

Yijk=μ+CPLi+Bj+CPLi×Bj+eijk,

where Yijk represents the responses of lamb k on crude protein level i to breed j , μ represents the mean, CPL i represents the crude protein level effect ( i is low or high), Bj represents the breed effect ( j is BB, QFO or NT), CPL i × Bj represents the interactions between crude protein level and breed, and eijk represents the residual error.

Differences between groups were evaluated by Duncan's multiple range test (DMRT); significance was declared at P<0.05 .

## 3 Results and discussion

The interactions were not presented in the tables because they did not have significant effects on any studied parameters.

### 3.1 Meat chemical composition

The chemical composition of meat was reported in Table 1. The moisture content of lamb meat was affected by breed ( P<0.05 ), which confirmed results by Ablikim et al. (2016) but not those of Suliman et al. (2021) on the effect of breed on meat moisture. This component was not affected by the diet's CP level; however, the meat lipid and protein contents were affected by this dietary treatment, with lower lipid ( P = 0. 05) and higher protein contents for the LP compared to HP groups ( P = 0. 08). This result could prove that lamb diets containing 11 % CP resulted in leaner meat than HP diets (16 % CP).

**Table 1:** Moisture (%) and chemical composition (percent of dry matter) of lamb meat.

	Breed^1^			Diet^2^		p value^3^	
	BB	QFO	NT	LP	HP	Breed	diet
Moisture	74.93^a^	73.31^b^	74.19^ab^	74.62	73.69	0.04	0.08
Ash	3.85	3.57	3.65	3.77	3.60	0.07	0.09
Proteins	71.36^a^	67.33^b^	71.28^a^	71.12	69.28	0.04	0.08
Lipids	19.21^b^	24.34^a^	19.45^b^	19.34	22.06	0.02	0.05

^1^ BB = Barbarine; QFO = Queue Fine de l'Ouest; NT = Noire de Thibar. ^2^ LP: low-protein diet; HP: high-protein diet. ^3^ Breed: breed effect; Diet: diet effect. ^a,b^ Means within rows with different superscripts differ significantly ( P<0.05 ).

The content of meat fat was significantly ( P<0.02 ) affected by the breed, being higher for the thin-tailed QFO (24.3 %) breed than for the fat-tailed BB or the thin-tailed NT breeds which had similar values (Table 1). This result confirmed the breed effect on lipid content shown by other researchers (Kuchtik et al., 2012; Yousefi et al., 2012). However, Echegaray et al. (2021b) recorded no significant ( P>0.05 ) differences in meat chemical composition between animals from different breeds. The protein content was higher for the fat-tailed BB and thin-tailed NT breeds than for the thin-tailed QFO breed (Table 2). So, meat from the fat-tailed breed had a higher protein content than one thin-tailed breed (QFO) but a similar content to the other one (NT). The breed effect on the protein was not shared by different authors. In some studies, using lighter lambs (37 and 34 kg), there were no differences (Yousefi et al., 2012; Suliman et al., 2021), while other authors indicated significant effects of breed on protein content in meat when using selected lighter lambs (Kuchtik et al., 2012). The between-breed differences in meat lipid and protein contents are probably related to genetic factors as the genetic selection may lead to improving carcass leanness. For the current study, the BB and NT breeds could be considered meat breeds with a high genetic potential to produce lean meat.

**Table 2:** Water cooking loss (WCL), pH and colour parameters of lamb meat.

	Breed^1^			Diet^2^		p value^3^	
	BB	QFO	NT	LP	HP	Breed	diet
WCL (%)	14.5^a^	13.2^a^	9.95^b^	12.2	12.6	0.01	0.73
pH 1	7.57^a^	7.36^b^	7.40^b^	7.49	7.44	0.01	0.73
pH 24	5.81^b^	5.91^a^	5.89^a^	5.84	5.90	0.02	0.09
d -pH	1.76^a^	1.45^b^	1.51^ab^	1.65	1.54	0.07	0.23
L	40.1	40.4	40.1	40.72	39.60	0.94	0.29
$a^{*}$	16.6^b^	16.7^b^	18.1^a^	17.1	17.3	0.15	0.71
$b^{*}$	6.77^b^	8.70^a^	7.84^ab^	7.48	8.03	0.11	0.46
C	18.0^b^	17.9^b^	19.8^a^	18.3	18.9	0.07	0.48
H	22.1	24.5	23.3	23.5	23.1	0.21	0.70

^1^ BB = Barbarine; QFO = Queue Fine de l'Ouest; NT = Noire de Thibar. ^2^ LP = low-protein diet; HP = high-protein diet. ^3^ Breed = breed effect; Diet = diet effect. ^a,b^ Means within rows with different superscripts differ significantly ( P<0.05 ).

### 3.2 The meat pH, colour and water cooking loss

The pH, colour and WCL values are summarized in Table 2. For all groups, the pH recorded 24 h after slaughter was about 5.8, being in the recommended levels to avoid meat quality deterioration (Majdoub-Mathlouthi et al., 2013; Ramírez-Retamal and Morales, 2014). The initial and ultimate pH values of meat were not affected by CP level (Table 2). This similarity resulted from similar energies for both diets and suggests that heavy lambs displayed the same glycogen muscle content (Chauhan et al., 2019). The ultimate meat pH was significantly affected by the breed ( P = 0.02), which confirmed previous results about the breed effect on meat pH (Kuchtik et al., 2012; Suliman et al., 2021).

The meat of the fat-tailed BB breed had the lowest pH (5.81) and registered the most important pH drop (Table 2). The differences in the meat pH observed between breeds may be attributed to differences in glycogen levels in muscles and pre-slaughter stress (Suliman et al., 2021). So, the actual results on pH hypothesized that BB was the less sensible breed for the transport conditions and slaughter process. The pH drop throughout carcass refrigeration is the natural consequence of the decrease in glycogen levels with the rigor mortis process. The post-mortem differences in pH decline between breeds were recorded, and some genotypes had greater decline in muscle pH than others (Chauhan et al., 2019).

The colour of meat is the most apparent quality criterion on which the consumer's meat purchasing decision is affected. Our results have not shown any significant effect of dietary treatment, in this case the CP level, on colour components, namely lightness ( $L^{*}$ ), redness ( $a^{*}$ ) and yellowness ( $b^{*}$ ). Also, there were no significant differences in these parameters among the three sheep breeds, except the yellowness ( $b^{*}$ ), which tended to be higher for the QFO breed. These results confirmed other results (Suliman et al., 2021) showing no differences in meat colour attributes between genotypes raised and slaughtered under the same conditions regardless the diet treatments when animals were slaughtered at similar ages and body weights, which is the case in the current study. The slightly higher value of yellowness ( $b^{*}$ ) for the QFO breed could be related to its higher lipid content. It was shown that $b^{*}$ is proportional to meat fat content; in fact, adipocytes are the natural cavities of lipid and carotenoid pigment storage (Hajji et al., 2019). So the more fat the meat contains, the yellower it will be. $L^{*}$ and $a^{*}$ colour parameter values equal to or exceeding 9.5 and 34, respectively, are acceptable by consumers for sheep meat (Khliji et al., 2010); the values recorded in the current study are in these recommended norms, since $L^{*}$ averaged 40 and $a^{*}$ averaged 17, regardless of diet CP level or breed type of lambs. The diet CP level did not have any significant effects on chroma or hue angle, while the breed tended to affect the chroma index, being higher for the NT breed than for the others (QFO and BB).

The water cooking loss (WCL) was not affected by the CP level, while the genotype affected ( p<0.001 ) this parameter (Table 2), which confirmed reported results on the breed effect on water-holding capacity (Kuchtik et al., 2012; Chauhan et al., 2019). This effect could be caused by the difference in the ultimate pH. It was shown that a low ultimate pH led to meat proteins with great cooking loss, while low cooking loss was generated by a higher ultimate pH (Suliman et al., 2021). In fact, the fat-tailed BB with the lowest pH had the greatest water cooking loss. On the other hand, Chauhan et al. (2019) explained the WCL difference between breeds by the toughness, given the relationship between the cooking loss and Warner–Bratzler shear.

### 3.3 Meat fatty acid profile and lipid indices

The meat fatty acid profile is presented in Table 3. The sum of palmitic, stearic and oleic FA accounted for around 86 % of the total fatty acids for all groups. This prevalence is in line with values commonly accepted for FAs of thin-tailed (Santos-Silva et al., 2002; Kuchtik et al., 2012; Carneiro et al., 2021;) and fat-tailed sheep (Ben Abdelmalek et al., 2020; Yagoubi et al., 2020). There were no significant differences among breeds and protein levels for this sum. The dietary CP level had affected linoleic fatty acid (C18:2 n-6tt ) concentration, which was higher for the low-protein level than the high-protein level (Table 3). These results were not expected since the diets were not divergent and have the same energy level and the same particle texture, resulting in the same amount of precursors of C18:3 n-3 and C18:2 n-6 . However, it was suggested that dietary CP regulated lipid anabolism and catabolism via modulation of the mRNA levels of key regulatory enzymes and fatty acid transport proteins in different muscle tissues and may modulate the FA profile (Wang et al., 2018). With different protein sources in isoproteic diets, Atti et al. (2005) found no difference in FA profile of lamb meat from the BB breed. Some research has shown that the protein level of the diet had an effect on the proportion of some FAs (C14:0, C16:0, C18:1, C18:2, C20:4) and concluded that the effect of protein level on IMF FA composition is difficult to interpret and needs more investigation for ruminants (De Smet et al., 2000). For pigs, recent studies tried to explain the mechanism of how dietary CP levels affect protein and fat metabolism, in order to provide adequate nutritional strategies that improve meat quality (Wang et al., 2018).

**Table 3:** Fatty acid profile of IMF^*^ in lamb meat (percent of total FA).

	Breed^1^			Diet^2^		p value^3^	
	BB	QFO	NT	LP	HP	Breed	Diet
C10:0	0.128^b^	0.164^a^	0.125^b^	0.137	0.133	0.001	0.70
C12:0	0.070^a^	0.068^a^	0.059^a^	0.062	0.068	0.40	0.50
C14:0	1.843^b^	2.155^a^	1.770^b^	1.89	1.88	0.04	0.94
C15:0	0.339^a^	0.295^a^	0.306^a^	0.33	0.29	0.52	0.12
C16:0	24.09^b^	26.85^a^	24.21^b^	25.02	24.44	0.001	0.29
C16:1 n-7	0.575	0.830	0.821	0.73	0.72	0.10	0.91
C17:0	1.509	1.225	1.401	1.52	1.25	0.37	0.07
C17:1	0.897	0.880	0.888	0.93	0.83	0.97	0.13
C18:0	17.58^a^	14.56^b^	17.83^a^	16.92	17.07	0.001	0.88
C18:1 n-9c	43.43	45.98	45.71	44.73	45.08	0.10	0.61
C18:1 n-7	1.155	0.962	0.991	1.01	1.09	0.08	0.37
C18:2 n-6tt	0.205	0.171	0.211	0.22^a^	0.16^b^	0.53	0.04
C18:2 n-6cc	3.490^a^	2.322^b^	2.394^b^	2.75	2.85	0.001	0.96
C18:3 n-3	0.341^a^	0.245^b^	0.257^b^	0.27	0.30	0.002	0.40
C18:2 c9-t11 (CLA)	0.265	0.214	0.244	0.238	0.254	0.155	0.47
C20:0	0.088	0.062	0.089	0.080	0.086	0.19	0.61
C22:0	0.135^a^	0.081^b^	0.068^b^	0.09	0.104	0.001	0.51
C20:4n6	1.236^a^	0.832^b^	0.809^b^	0.91	1.06	0.01	0.36
C20:5 n3	0.153^a^	0.030^b^	0.027^b^	0.08	0.062	0.05	0.52
C22:4 n-6	0.129^a^	0.088^b^	0.096^ab^	0.099	0.177	0.04	0.26
C22:5 n3	0.266^a^	0.132^b^	0.136^b^	0.164	0.212	0.001	0.35
C22:6 n-3	0.055^a^	0.014^b^	0.016^b^	0.0311	0.0314	0.02	0.85

^*^: intramuscular fat. ^1^ BB = Barbarine; QFO = Queue Fine de l'Ouest; NT = Noire de Thibar. ^2^ LP = low-protein diet; HP = high-protein diet. ^3^ Breed = breed effect; Diet = diet effect. ^a,b^ Means within rows with different superscript differ significantly ( P<0.05 ).

For the current study, a majority of the individual FA percentages were affected by the breed. The genotype effect on FA profile was confirmed (Yousefi et al., 2012; Turner et al., 2014). The C10:0 and, consequently, the SCFA were higher ( P<0.001 ) for QFO than other breeds. The same tendency was recorded for C14:0 and MCFA ( P<0.04 ). The stearic FA was also higher ( P<0.001 ), while the palmitic FA was lower ( P<0.001 ) for this thin-tailed breed compared to the other thin-tailed (NT) and fat-tailed (BB) breeds. These tendencies resulted in the same sum of SFA proportion for all breeds (Table 4). The oleic FA value was higher for both thin-tailed breeds compared to the fat-tailed one ( P<0.10 ), while linoleic (C18:2 n-6 ) and linolenic (C18:3 n-3 ) FAs, as well as all identified PUFAs, were higher ( P<0.001 ) for the fat-tailed breed compared to the thin-tailed ones. This result conforms with other findings on FA profile and fat content for thin-tailed lambs (Yousefi et al., 2012). Moreover, the FA composition of the BB breed meat showed a relatively high PUFA level, which is beneficial to human health by reducing the risk of atherogenicity, thrombogenicity and hypercholesterolemia, which confirmed the last conclusion related to the macro-composition of the BB meat as lean meat regarding its higher protein and lower fat contents than the QFO breed.

The concentration of C18:1 *cis*-9 in the current study (superior to 43 %) is high compared to the usual values; this may be due to breed precocity and adult weight. The C18:1 *cis*-9 proportion was 32 % for Awassi and Turkish × Awassi (Çelik and Yilmaz, 2010); it was also lower for Suffolk and Katahdin lambs slaughtered at 40 and 27 kg, being 27 % and 35 %, respectively (Turner et al., 2014). The breeds with heavy adult weights are generally less fat and may contain less C18:1 *cis*-9. Tunisian sheep breeds are precocious, so their carcasses contain more fat tissues and their meat more C18:1 *cis*-9. The high content of C18:1 *cis*-9 in the current study could also be explained by the lamb's age at slaughter (12 months) at heavy weight, which promotes the accumulation (about 50 %) of intermuscular fat (Hajji et al., 2016), given this value was around 35 % for young BB lambs slaughtered at around 45 kg (Majdoub-Mathlouthi et al., 2013). The high concentrations of docosapentaenoic acid (DPA) (C22:5 n-3 ) and docosahexaenoic acid (DHA) (C22:6 n-3 ) in the BB breed are in synergy with the results by Raes et al. (2001) on the double-muscled Belgian Blue genotype, accumulating more subcutaneous fat and less internal fat, which is also the case for the BB sheep having a high (37 %) subcutaneous fat (Hajji et al., 2016), resulting in higher conversion of C18:3 n-3 to C20:5 n-3 and C22:5 n-3 but not C22:6 n-3 . The sum of PUFA was higher for fat-tailed BB (Table 4) than for both thin-tailed breeds. Globally, the meat of the BB breed contains more beneficial PUFAs, while the QFO breed had the richest meat in SFAs. In addition, the BB breed had the highest sums of n-3 and n-6 PUFAs and the highest PUFA / SFA ratio (0.135 vs. 0.091), while the n-6/n-3 ratio was similar for all breeds (Table 4), averaging 8. This ratio (PUFA n-6/n-3 ) is considered an important indicator for the nutritional value of fats in foods. In the current study, it is superior to the dietetic value (4.5) recommended by the UK Department of Health (Great Britain, Department of Health, 1994) for human health. Some health problems, particularly coronary heart disease, were correlated with high SFA and low n-3 PUFA consumption (Russo, 2009). The low PUFA / SFA and high n6/n3 ratios reported in the current study can be explained by the high level of concentrate in the diet ( 2/3 ) and the high live weight of animals (51 kg). It was reported that an increasing weight of concentrate resulted in a high PUFA n-6/n-3 ratio of meat (Santos-Silva et al., 2002; Aksoy et al., 2019; Gruffat et al., 2020). In addition, higher levels of meat C18:2 n-6 than C18:3 n-3 may be due to a reduced bio-hydrogenation in rumens. This occurs when the form of the diet is similar, particularly in typical diets based on concentrates that are rich in C18:2 n-6 , which is the case for the actual study (Meeprom et al., 2019).

**Table 4:** Sums of fatty acids and nutritional indices in lamb meat.

	Breed^1^			Diet^2^		p value^3^	
	BB	QFO	NT	LP	HP	Breed	Diet
SFA	45.79	45.47	45.85	46.08	45.33	0.93	0.37
SCFA	0.13^b^	0.17^a^	0.12^b^	0.144	0.135	0.001	0.38
MCFA	2.25	2.52	2.13	2.29	2.23	0.11	0.70
LCFA	43.41	42.78	43.58	43.64	42.96	0.71	0.37
∑ MUFA	46.51	48.77	48.48	47.59	48.02	0.14	0.56
∑C18:1	45.04	47.06	46.77	45.92	46.46	0.21	0.50
∑ PUFA	6.14^a^	4.06^b^	4.19^b^	4.76	5.11	0.006	0.83
∑n6	5.085^a^	3.42^b^	3.54^b^	4.03	4.21	0.002	0.88
∑n3	0.66^a^	0.39^b^	0.41^b^	0.47	0.54	0.001	0.32
Total CLA	0.265	0.214	0.244	0.238	0.254	0.155	0.47
DFA	70.27^a^	67.39^b^	70.53^a^	69.35	70.17	0.002	0.23
n6/n3	7.70	8.77	8.63	8.57	7.80	0.40	0.13
PUFA / SFA	0.135^a^	0.089^b^	0.092^b^	0.105	0.112	0.001	0.67
AI	0.604^b^	0.674^a^	0.596^b^	0.626	0.606	0.05	0.37
TI	1.554	1.591	1.602	1.601	1.558	0.43	0.61
HPI	1.655^a^	1.483^b^	1.678^a^	1.598	1.649	0.05	0.56
h/H	1.911^a^	1.725^b^	1.921^a^	1.839	1.907	0.05	0.41

^1^ BB = Barbarine; QFO = Queue Fine de l'Ouest; NT = Noire de Thibar. ^2^ LP = low-protein diet; HP = high-protein diet. ^3^ Breed = breed effect; Diet = diet effect. DFA: desirable fatty acids, i.e. C18:0 + unsaturated fatty acids; AI: atherogenic index, i.e. (C12:0 + 4 C14:0 + C16:0) / ( ∑ PUFA + ∑ MUFA); TI: thrombogenic index, i.e. (C14:0 + C16:0 + C18:0) / [( 0.5×Σ MUFA) + ( 0.5×Σn6 PUFA) + ( 3×Σn3 PUFA) + ( n3/n6 )]; HPI: health-promoting index =Σ UFAs / C12:0 + 4 × C14:0 + C16:0. h/H : hypocholesterolemic / hypercholesterolemic ratio = (*cis*-C18:1 + Σ PUFA) / (C12:0 + C14:0 + C16:0). SCFA: short-chain fatty acid. MCFA: medium-chain fatty acid. LCFA: long-chain fatty acid. n6: omega-6 fatty acid. n3: omega-3 fatty acid. CLA: conjugated linoleic acid. ^a,b^ Means within rows with different superscripts differ significantly ( P<0.05 ).

In addition to PUFA, SFA, the ratio PUFA / SFA and the ratio n-6/n-3 , the FA composition permits the calculation of two other indices in relationship with the atherogenic and thrombogenic power of food, which is very important in human health; they are expressed by AI and TI, respectively. The AI was not affected by the dietary treatment. However, the meat of BB and NT breeds had lower AI than that of the QFO breed; this result has parallels with the variation of undesirable FA (C12, C14 and C16), which had a higher sum in QFO meat. Overall, the AI was less than the limit threshold (1), which was suggested for dietetic food (Ulbricht and Southgate, 1991) and then reported in many studies (Majdoub-Mathlouthi et al., 2015; Gruffat et al., 2020). The HPI, calculated as the reverse of AI, was lower in the meat of the QFO breed. In relation to the thrombogenic aspect, the TI was similar among all groups (averaging 1.6), without any effect on sheep breed or diet. This value for meat of heavy (50 kg) and aged (1 year) sheep is higher than the limit of recommended values of TI (1.1) for human health as previously reported (Majdoub-Mathlouthi et al. 2015; Gruffat et al., 2020; Carneiro et al., 2021) for meat of younger and lighter lambs (5–6 months, 26 kg), broiler meat (0.7; Attia et al., 2017), and fish (0.3; Biandolino et al., 2023). Another explanation of the higher values for TI of meat of lambs from the current study, reared in a feedlot, is that this rearing mode resulted in higher TI than grazing mode (Majdoub-Mathlouthi et al., 2015; Gruffat et al., 2020). Ulbricht and Southgate (1991) consider the myristic, palmitic and stearic FAs (C14:0, C16:0 and C18:0) as thrombogenic; hence, the TI values should have the same tendency as the sum of these FAs. This is how the TI established in the current study was higher than those of Gruffat et al. (2020) and Carneiro et al. (2021), where the sum of the thrombogenic FA was lower (40 % vs. 43 %). Therefore, it could be concluded that the meat issued from heavy and aged lambs with high TI, which is the case for the current study, should cause some health risks (coronary heart disease) for humans.

**Table 5:** Sensory attributes of lamb meat.

	Breed^1^			Diet^2^		p value^3^	
	BB	QFO	NT	LP	HP	Breed	diet
Tenderness	6.28	6.62	6.10	6.20	6.44	0.25	0.35
Juiciness	6.33	6.20	6.09	6.25	6.156	0.77	0.72
Flavour	7.39^a^	6.77^b^	6.59^b^	6.87	6.96	0.01	0.72
Acceptability	6.78^a^	6.22^b^	6.17^b^	6.31	6.47	0.09	0.52

^1^ BB = Barbarine; QFO = Queue Fine de l'Ouest; NT = Noire de Thibar. ^2^ LP = low-protein diet; HP = high-protein diet. ^3^ Breed = breed effect; Diet = diet effect. ^a,b^ Means within rows with different superscripts differ significantly ( P<0.05 ).

Moreover, another interesting way to evaluate the nutritional value of the meat based on its FA profile could be the use of ratios established on functional effects of FAs, like the ratio hypocholesterolemic / hypercholesterolemic ( h/H ) which expresses the effects of individual FAs on the metabolism of cholesterol; the indicated value is 2 (Santos-Silva et al., 2002). For the current study, the h/H ratio of meat was not affected by the sheep diet, given that all animals received the same regimen composition based on concentrate, while dietary differences in h/H were shown between meat of lambs reared on pasture vs. concentrate (Santos-Silva et al., 2002). However, significant differences ( P<0.05 ) were observed between breeds with a higher value for meat of BB and NT than QFO (1.9 vs. 1.7). For the first breeds, the h/H value is close to the recommended and confirmed value for meat of lighter lambs (24–30 kg) from thin-tailed lamb breeds (Santos-Silva et al., 2002; Carneiro et al., 2021) and some fish (2.4; Biandolino et al., 2023). Hence, according to results on h/H recorded for BB and NT breeds, their FA profile could be considered hypocholesterolemic. Furthermore, this result is confirmed for BB breed by the highest C20:4 n-6 content (1.236 vs. 0.820), which has in vitro cholesterol-lowering attributes (Viljoen, 1999). However, higher h/H ratios are associated with a greater recognition of foods as beneficial, as demonstrated with the common carp (*Cyprinus carpio* L.), where the h/H values exceed 4 (Ivanova and Hadzhinikolova, 2015).

### 3.4 Sensory attributes

The CP level of the diet had no effect on meat sensory traits; the panellists had evaluated the meat as medium tender, juicy and acceptable (Table 5). The meat of different breeds was evaluated as having the same tenderness, juiciness and acceptability, which confirmed results by Suliman et al. (2021) on the similarity of these sensorial traits between breeds. However, Arshad et al. (2018) have shown that meat flavour is affected by many factors, particular diet. For tenderness, our results confirmed the absence of differences among breeds on tenderness, mechanically measured as anteriorly reported (Ablikim et al., 2016). However, other authors reported significant effects of breed on sensorial traits (Chauhan et al., 2019; Suliman et al., 2021). Hence, these non-similar results should depend on compared breeds and production systems. On the other hand, it was shown that meat tenderness (measured as shear force) was correlated to meat intramuscular fat and cooking loss (Ablikim et al., 2016), while in the current study, despite the significant effect of breed on these meat attributes, there was no breed effect on tenderness. The unique sensorial trait affected by breed was the flavour, and the meat of the BB fat-tailed breed had the highest flavour note (Table 5). This observation is related to the abundance of PUFAs in BB meat; the effects of the FA profile on meat flavour were shown by several authors (Arshad et al., 2018; Renna et al., 2019). In comparison with the meat of both thin-tailed breeds (QFO and NT) containing more SFAs, the higher PUFA value of BB needs a lower temperature to be melted, so it has a higher chance to release volatile molecules during cooking through several chemical reactions including the Maillard reaction and lipid oxidation (Shahidi and Hossain, 2022).

## 4 Conclusions

The meat of young sheep finished in feedlots on a high-energy diet with varying protein levels exhibited comparable quality traits and fatty acid (FA) profiles. However, genetic factors, particularly breed, significantly influenced these traits. The meat from the fat-tailed Barbarine (BB) breed was notably richer in proteins and polyunsaturated fatty acids (PUFA), with a higher PUFA / SFA ratio compared to thin-tailed breeds. The meat produced by these heavy sheep, typically consumed by southern Mediterranean populations during ceremonies, is characterized by higher fat content, with FA ratios ( n6/n3 , PUFA / SFA) and lipid indices, particularly the thrombogenic index, falling outside the recommended ranges for human health. Nevertheless, the Barbarine fat-tailed breed stands out with more favourable lipid indices, including the atherogenic index (AI) and the hypocholesterolemic / hypercholesterolemic ( h/H ) ratio. These results suggest that, in terms of lipid profile, the meat of the BB breed may be considered hypocholesterolemic and a potentially healthier option for consumers, despite the high fat content.

## Data Availability

The original data of the paper are available upon request to the corresponding author.

## Code availability

The software code of the paper is available upon request to the corresponding author.
